# Towards an Architecture to Guarantee Both Data Privacy and Utility in the First Phases of Digital Clinical Trials

**DOI:** 10.3390/s18124175

**Published:** 2018-11-28

**Authors:** Fabio Angeletti, Ioannis Chatzigiannakis, Andrea Vitaletti

**Affiliations:** Department of Computer, Control, and Management Engineering “Antonio Ruberti”, Sapienza University of Rome, 00185 Rome, Italy; angeletti@diag.uniroma1.it (F.A.); vitaletti@diag.uniroma1.it (A.V.)

**Keywords:** privacy protection, security, human-centered computing, mobile devices, IoT, survey, performance evaluation

## Abstract

In the era of the Internet of Things (IoT), drug developers can potentially access a wealth of real-world, participant-generated data that enable better insights and streamlined clinical trial processes. Protection of confidential data is of primary interest when it comes to health data, as medical condition influences daily, professional, and social life. Current approaches in digital trials entail that private user data are provisioned to the trial investigator that is considered a trusted party. The aim of this paper is to present the technical requirements and the research challenges to secure the flow and control of personal data and to protect the interests of all the involved parties during the first phases of a clinical trial, namely the *characterization* of the potential patients and their possible *recruitment*. The proposed architecture will let the individuals keep their data private during these phases while providing a useful sketch of their data to the *investigator*. Proof-of-concept implementations are evaluated in terms of performances achieved in real-world environments.

## 1. Introduction

In the last decade, we witnessed a tremendous progress towards the interconnection of the digital and physical domains, giving rise to the “Internet of Things” (IoT). The anticipated exponential increase of interconnected devices paved the way for new systems that orchestrate myriads of devices, web services, business processes, people, companies, and institutions. A particular domain, where the coexistence and cooperation of embedded systems with our social life is unveiling a brand new era of exciting possibilities, is that of digital health [1]. With the ever-increasing amount of data that are inherent to an IoT world, drug developers can potentially access a wealth of real-world, participant-generated data that is enabling better insights and streamlined clinical trial processes.

Developing drugs is a challenging process. Only around one in 10 drugs in development (called Phase 1) actually makes it through to the market [2]. This low rate to enter the market is one factor contributing to the high costs of drug development. A recent study indicates that developing a drug from bench to market costs an estimated $2.6 billion [3]. A large portion of those costs is related to (a) the stage of recruiting an adequate number of patients and (b) retaining the patients throughout the trials. Currently, more than 244,000 studies are registered in the world out of which more than 42,000 are currently recruiting [4]. The need to access an appropriate pool of patients in order to execute clinical trials is well known to the broader public. Some of these studies require thousands of participants, each of whom must meet precise criteria to join. Given the strict qualification criteria imposed by the researchers, only about 5% of candidates eventually constitute the group participating in clinical trials. Therefore, it is not surprising that 80% of these studies are delayed due to recruitment problems, according to the Center for Information and Study on Clinical Research Participation (CISCRP) [5]. The same study [5] identified that 81% of responders consider clinical research studies “very important” to the discovery and development of new medicines and 80% of them would be willing to participate in a research study. Long recruitment phases prolong the execution of trials, thus increasing the time it takes for innovative new medicines to be studied and approved, leaving patients to wait years for new treatment options.

According to a 2012 online survey [6], 85% of the responders perceive privacy concerns as a barrier to share health information. It is clear that collected data may be used to extract or infer sensitive information about users’ private lives, habits, activities and relations, which all refer to individuals’ privacy [7,8]. About half of the responders were either concerned or very concerned about the re-identification of their anonymized health and medical information. If data were irreversibly anonymized, 71% of respondents were willing to share data with researchers. During a clinical trial recruiting phase, when the benefits of a possible future enrollment have not been fully clarified, patients expect that their medical condition information is kept confidential. It is, therefore, imperative to introduce new methods for facilitating recruitment that respects the privacy and confidentiality of the patients in order to maximize the participation of people—particularly in rare diseases where the communities of patients are small. A major problem arises: “Data sharing could put clinical trial participants at increased risk of invasions of privacy or breaches of confidentiality. As a result, participants could suffer social or economic harms” [9]. Therefore, recognizing that the ownership of data should be of the participants [10] is not enough. Even accepting that consumers must have control over their data and should receive a fair part of the value created by the companies using their data [11], the protection of personal data is not guaranteed. It is important to incorporate suitably selected cryptographic tools and blockchain technologies in combination with IoT technologies to provide a holistic environment that respects the privacy of personal data and guarantees its confidentiality. We need to deliver a more sustainable and responsible data economy, focused on delivering innovative and personalized services that better fit real consumers’ needs, contributing to enhance their lives and the society as a whole while always protecting their personal data [11].

During the execution of the trials, the collection of high-quality data is absolutely vital. For this reason, trial centers require regular tests and observations to be conducted at their premises in order to guarantee the accuracy of data collection. Interestingly, 70% of potential participants live more than two hours away from the nearest study center [4]. It is, therefore, common for patients to travel to those centers for regular tests and observations, sometimes several times each week for the duration of the trial. Such complexities sometimes overcome the perceived benefits of participating in a trial, inevitably increasing the attrition rate of patients.

Understanding the above issues and addressing them adequately is critical in developing successful digital health solutions. As technology becomes more accessible and affordable, the role of digital health data will become vital in clinical trials. It is well known that smartphones are a ubiquitous technology—in 2015, almost two-thirds of people in the U.S. owned a smartphone and almost half owned a tablet [12]. During the same year, about 300 clinical trials were reported to involve wearable technology [13]. According to a Business Insider Estimates study in 2015, more than 161 million healthcare devices will be installed by 2020.

**Figure 1:**
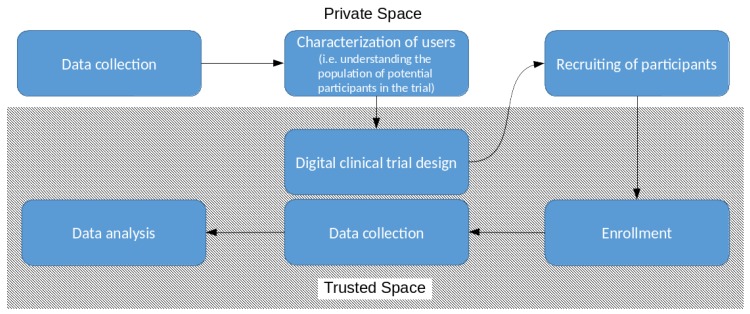
A simplified representation of the main phases for conducting digital clinical trials.

In this work, we consider the process of conducting digital clinical trials depicted in Figure 1 focusing on the *data collection*, *characterization of users*, and *recruiting of participants* phases. We consider the *private space* of the potential participants to the trial and the *trusted space* of the *investigator*, namely the entity that actually conducts the trial. Data in the private space should be kept private until the candidate is not actually enrolled in the trial becoming a participant. This participation will hopefully provide some benefits to the user and consequently s/he will finally have the necessary incentives to disclose her/his data to the investigator. On the other end, the first need of the investigator is the *characterization of the population* of users. The investigator needs to know the amount of users potentially interested in participating (and fit to participate) in the digital clinical trial. It is desirable to have some statistical knowledge on their personal data on their health and habits (possibly augmented by IoT devices) and take into account insights from previous trials. In this phase of the clinical trials, the main issue is how to guarantee the trade-off between the privacy of the users and the utility of the data for the investigator. This step allows the investigator to evaluate if there is a critical mass of potential participants to start a trial and thus proceeding with the *trial design*. Once the trial has been designed, during the *recruiting phase*, patients matching the needs for the designed trial and willing to participate are recruited. These patients are required to enroll upon accepting the informed consent. Enrolled patients give their previously collected data to the investigator that can finally analyze it. This process will leverage the ability of modern technologies to communicate over the Internet in order to (a) reach nearly an unlimited number of potential participants and (b) collect relevant data at home without requiring participants to regularly visit the “study centers.”

**Structure of the paper.** In Section 2, we introduce the use cases that will drive the design of our solutions. We dive into the concept of privacy and the implications of recent regulations (such as HIPAA and GDPR) in conducting IoT-assisted digital clinical trials in Section 3. In Section 4, we illustrate the common technical challenges posed by the use of IoT in healthcare. Section 5 shows the current approach to handle personal health data demanding all the privacy issues to the trusted space (i.e., to the investigator). In Section 6, we present our solution in which the data collection, the characterization of the users, and the recruitment of the participants are managed in the private space, trying to protect the interests of both the investigator (i.e., the utility of the data) and the participants (i.e., the privacy of the data). Finally, in Section 7, we present the state of the art on IoT technologies in healthcare and clinical trials in particular, and, in Section 8, we highlight the main contributions of this paper and propose some future steps to extend this work.

## 2. Use Cases

In this section, we present the use-cases that help us to capture the key aspects of the characterization and and recruitment phases (see Figure 1).

### 2.1. Characterization of the Users

The *investigator* is interested in grouping candidates according to their characteristics in order to better design the clinical trial. For this purpose, this investigator starts the *data clustering phase* by carefully specifying the desired features relevant for the purpose of the trial. The features include the evaluation of specific biometric attributes (e.g., body composition, heart operation, daily activity, etc.) collected from the patient using wearable technologies over a given period of time (e.g., blood pressure for past week, etc.). The *investigator* has conducted certain accuracy evaluation tests over various off-the-shelf wearable devices and smart devices and has compiled a list of trusted devices that it considers accurate enough so that data collected from these devices can be used through the digital screening phase. Based on an adequate set of genuine historic values collected from one or more of these trusted devices, a privacy-preserving clustering algorithm is executed to allocate patients in groups (clusters) of similar characteristics (based on the defined features). This information is finally made available to the *investigator* that can use it for the purpose of the *recruting phase*. We translate these steps into the following requirements.

  R1 The quality of data provided by participants is guaranteed (only consider data collected from approved devices).
R2C Candidates are clustered on the bases of the results of a well-defined *clustering algorithm* automatically executed over a provided data set of historic values.
  R3 The candidate must provide proof that the historic data set is real and collected over the stated period of time.

An individual participating to this phase expects that the privacy of her/his personal data will be respected and the confidentiality of the private data will be guaranteed. The system is secure enough to ensure that only devices installed by the individual can participate and that no fake data can be injected into the system.

  R4 Data are collected by certified and trusted devices.
  R5 Fake data cannot be introduced into the private space.
R6C Candidates’ privacy is preserved during the clustering phase. Namely, none of the users’ data are disclosed to the institute. The only information received back from the institute are the ones necessary to identify the clusters without disclosing data on the single users.
  R7 Periodic certificates are provided to prove the authenticity, integrity, and conformance of collected data (see R3).

### 2.2. Patient Recruitment

When the patients are selected, possibly in view of the characteristics identified in the data *clustering phase*, the investigator is ready to design the trial and proceed to the next phase by recruiting specific patients. We assume that the *investigator* starts the patients’ *recruiting phase* by carefully specifying the desired patient profiles and the digital screening process. Furthermore, the screening relies on the evaluation of specific biometric attributes collected from the patient using wearable technologies over a given period of time. We translate these steps into the following requirements.

  R1 Same as that in the data clustering phase.
R2R The inclusion/exclusion of a candidate is based on the execution of a well-defined *recruiting test* automatically executed over the provided data set of historic values. As an example, the recruiting test can be the distance of a user from a given centroid identified during the *data clustering phase*.
  R3 Same as that in the data clustering phase.

An individual that wishes to be considered for a specific clinical trial expects that the privacy of her/his personal data will be respected and the confidentiality of the private data will be guaranteed. If during the digital screening phase the individual is excluded, then the digital health system should guarantee that no personal data have been retained by the *investigator*. The system must be secure enough to ensure that only devices installed by the individual can participate and that no fake data can be injected into the system.

  R4 Same as that in the data clustering phase.
  R5 Same as that in the data clustering phase.
R6R Candidates’ privacy is preserved during the recruiting phase. The recruiting test is privacy-preserving, namely, it does not disclose patient’s data to the institute. Data never leave the private space of the patient unless the candidate voluntarily enrolls in the trial because s/he is eligible according to the outcome of the recruiting test (see R2).
  R7 Same as that in the data clustering phase.

Once in the enrollment phase, the data are eventually delivered to the *investigator*; consequently, the participant has to trust the investigator for the successive management of her/his data. Huge and important markets, such as one of the digital media, have fundamentally failed to design data protection mechanisms capable of avoiding the duplication of data or their illegitimate sharing to the wider audience. However, the scenario we consider in this work is fundamentally different: while in digital media markets the data receivers are all potential Internet users, in the clinical trial case, the intended receiver is an *investigator* with a good reputation.

## 3. Privacy

We leave around our digital traces using modern ICT applications [14]. These traces are collected, assembled, and used in uncountable ways that often are nonetheless difficult to imagine. There are various reports of concerns regarding the violation of privacy, with particular emphasis on information privacy [15,16]. It is not possible to avoid all data collectors and in particular those services that can only be accessed by giving up some personal information [17]. On most websites, applications, or services, the disclosure of personal information allows access to premium features, gifts, enhancements in the online experience, and much more. Paradoxically, the benefits in terms of services offered have such a big value that a significant number of people are willing to give up their privacy for convenience [15,17,18,19,20]. Online users show privacy concerns about the usage, the disclosure, and the protection of their personal health information [21,22]. They are also sensible to the fact that it is possible that undesirable social and economic consequences can happen following a misuse of such data [23]. It is, therefore, necessary to maintain the privacy of information collected during healthcare processes because of significant economic, psychologic, and social harm that can come to individuals when personal health information is disclosed [24,25]. There are multiple definitions of privacy [26,27,28], each one focused on different declinations of the same principle: *“the ones right to manage valuable personal information”.* Certain studies account for some critical points regarding privacy: improper access, unauthorized use (both direct or secondary), errors, and the collection of personal information [29,30,31,32,33]. Information privacy raises issues of access control (user authentication and authorization) and the need for data authentication. In a digital health system, all information is converted into a digital form. Therefore, data protection and privacy protection are very closely connected. In this sense, the goal of security is the application of cryptographic protocols for data transmission and storage.

A healthcare information security system should be designed to guarantee the following [24,34,35].

1. The privacy of patients and the confidentiality of health care data (prevention of unauthorized disclosure of information).
2. The integrity of healthcare data (prevention of unauthorized modification of information).
3. The availability of health data for authorized persons (prevention of the unauthorized or unintended withholding of information or resources).

### 3.1. The Role of Trust

The research is well aware of the concern of privacy about the health information of individuals [36,37,38,39,40]. As part of the Health Insurance Portability and Accountability Act (HIPAA), introduced in 1996, a huge step in the handling and protection of sensible health information was made. Additionally, it brought to the forefront some privacy concerns [41]. These studies indicate that the lack of *trust* in ICTs and digital health care affects very seriously any effort to migrate from the conventional healthcare procedures to electronic systems. The term *“trust”* implies that the agreement depends on a third party (another person, institution, company, or other) based only on the belief of its integrity and/or benevolence [42,43,44]. The trustness has been the fundamental pre-requisite for the progress of commerce and prosperity in human societies [45] and determines to which extent an individual wants to depend on others.

The central role of trust as a major type of social capital in online activities is well established [45,46,47]. According to the above, any successful digital healthcare system should target at increasing a citizen’s trust. It is clear that both *trust* and *security* play central and fundamental roles: “The more people trust others, the less concern they have for misuse of personal information” [14]. As privacy is connected to security, a similar relationship is also observed between trust and security [48]. Trust, however, is difficult to establish in the digital health domain since it requires interactions between computers, between humans, and between humans and computers.

### 3.2. Data Protection Regulations

The need for protecting individuals’ privacy has been recognized by law enforcement agencies, leading to the creation of laws for data protection. The American Civil Liberties Union (ACLU) believes that a privacy policy for health information should be based on the following principles [24,49].

1. Strict limits on access and disclosure must apply to all personally identifiable health data, regardless of the form in which the information is maintained.
2. All personally identifiable health records must be under an individual’s control. No personal information may be disclosed without an individual’s uncoerced, informed consent.
3. Health-record information systems must be required to build-in security measures to protect personal information against both unauthorized access and misuse by authorized users.
4. Employers must be denied access to personally identifiable health information on their employees and prospective employees.
5. Patients must be given notice of all uses of their health information.
6. Individuals must have a right of access to their own medical and financial records, including rights to copy and correct any and all information contained in those records.
7. Both a private right of action and a governmental enforcement mechanism must be established to prevent or remedy wrongful disclosures or other misuses of information.
8. A federal oversight system must be established to ensure compliance with privacy laws and regulations.

A new European Union-wide framework known as the General Data Protection Regulation (https://ec.europa.eu/commission/priorities/justice-and-fundamental-rights/data-protection/2018-reform-eu-data-protection-rules_en) (GDPR) has been introduced that provides a more uniform interpretation and application of data protection standards across the EU. Essentially, it constitutes a fundamental change in the management of data privacy designed to protect and empower all EU citizens’ data privacy and with severe implications in the way organizations across the EU approach data privacy. While the purpose of the GDPR is to protect personal data at large, namely “any information relating to an identified or identifiable natural person”, in this paper the focus is on clinical trial data, as the collection and analysis of sensitive personal data (e.g., health data) is required.

The regulatory framework defines three main roles: **The Subject**, namely the resident or individual providing her/his data to the organization for the purpose of the clinical trial, **The Data Controller**, namely the *investigator* that determines the purpose and meaning of the processing (i.e., the clinical trial) of personal data provided by the subjects, and **The Data Processor**, who processes the personal data on behalf of the Data Controller. Note that in many cases the *investigator* has the double role of Data controller and Data Processor. The following is a short summary of the main requirements defined in the law enforcement directive.

- **Explicit Consent.** Clear and definite conditions for acquiring consent from data subjects (citizens) to process data.
- **Data Protection Officer.** A person is appointed to handle the necessary internal recordkeeping requirements.
- **Sanctions.** Non-compliance can result in serious penalties.
- **Territorial Scope.** The directive applies to all organizations processing data from data subjects (citizens) residing in the EU, not only EU-based organizations.
- **Right to Access.** The data subject shall have the right to obtain from the controller confirmation as to whether or not personal data concerning her/him are being processed, and, where that is the case, to access the personal data and some other information.
- **Right to Rectification.** Incorrect data has to be rectified.
- **Right to Be Forgotten.** Data subjects have the right to request data controllers to erase their data.
- **Data Portability.** Data subjects have the right to request their data in a portable format, which allows one to transfer its data to another data controller.
- **Data Protection by Design and by Default.** Develop default privacy protection mechanisms and implement monitoring processes.
- **Notification Requirements.** Data breaches must be reported without undue delay.

Clinical trial data are, however, a “special” data category, whereby processing is necessary for scientific or research purposes. This special data category negates the subject’s right to erasure or portability. This is due to the fact that clinical data cannot just be removed or transferred from a dataset, without affecting the audit trail or the statistical outcome. Subjects can, however, leave a trial to prevent additional data collection. In this context, the right of data portability means that clinical trial subjects have the right to receive their personal data in a commonly used and machine-readable format, and transmit such data to another organization.

Clinical trial providers must identify the data that is being processed, where it is transferred to, who processes the data, what it is used for, and any risks and processes and must ensure all employees are trained. Furthermore, they have to provide all this information to potential participants in a trial and keep records to show what individuals have consented to, what they were told, and when and how they consented. Note that a clinical trial provider is a processor from a customer perspective but also a controller of data in terms of personnel, sales, and sub-contractors. As a consequence, clinical trial companies have obligations to make sure that rules are in place and followed.

Particular interest is Article 32 of the directive that states that *“the controller and the processor shall implement appropriate technical and organizational measures to ensure a level of security appropriate to the risk”.* To this purpose, a crucial component of data collection in clinical trials is the distinction between pseudonymization and anonymization. Any pseudonymized data that can still be tied to an individual patient with the help of other information will still be considered personally identifying information (*PII*). Only fully anonymized data will lose the PII label, so trials must make the distinction between these two data types in trial protocols. A part of this work focuses on the technical implications of Article 32 in the context of clinical trials when IoT devices are employed at home to collect relevant health and personal data for the trial.

## 4. Data Collection in the IoHT

Today, multiple IoT devices for healthcare (namely *IoHT*) are available, from professional infusion pumps and ventilators to smartwatches and from portable blood analyzers to mobile EKG units. These are the devices used by the *investigators* to collect the necessary data to design and to conduct the digital clinical trials. From the work in [50], it is clear that IoT-generated data must retain some properties such as accuracy, freshness, and availability. The successful integration of IoT technologies in healthcare requires one to address very specific technical challenges that are briefly presented in this section.

The implementation and deployment of effective solutions able to properly address these challenges is crucial for the success of any real world clinical trial. In Section 6.2, we present the use of some “standard” cryptographic techniques (e.g., signature and hashing) to guarantee the accuracy, integrity, and authenticity of the data, and we also introduce the use of the blockchain as an emerging tool to guarantee some security properties of the data collected in the trial.

### 4.1. Accuracy

Heart rate, the glucose level in the blood, the number of steps made per hour, and the daily caloric intake are examples of information that can be gathered from IoHT devices. Like any other information, they have a certain accuracy that characterizes the data [51]. For example, it was studied [52] that heart rate monitoring made by common activity trackers and smartwatches have accuracies that range from 99.9 to 92.8%; thus, in certain scenarios they can be treated as accurate. In [53,54], the authors measured the performances of a very common activity band with respect to professional calorimeters. While during activities made on plane surfaces the accuracy was relatively high (>80%), a slight inclination change produced a notable degradation in performances, achieving a poor evaluation of burnt calories, with accuracy degrading by more than 40%. Another interesting aspect is that accuracies of different parameters can vary deeply depending on sensor positioning. As an example, sensors positioned on the wrist, on the chest, or on the hip achieve different accuracy [55].

### 4.2. Authenticity

There exist multiple entities that could generate data, so establishing an authentication method to verify the source of data and avoid poor quality data or tampered data is required. Users can be interested in faking data for multiple reasons. An example could be the assumption of opiate drugs that cause dependence [56]. A patient may be willing to fake data in order to receive stronger medications or additional doses of the same drug but for a longer period of time. An authentication method is therefore needed for all the IoT devices that participate in a digital clinical trial. A consistent number of works in the literature provide primitives for authentication in low power systems and, in general, in IoT devices. For example, a two-way authentication system based on a Datagram Transport Layer Security (DTLS) protocol could work [57], as could an authentication and access control framework based on the Constrained Application Protocol (CoAP) [58]. A certificate-based authentication mechanism for IoT can also be used in order to allow the sensor nodes and the end-users to authenticate each other and initiate secure connections [59].

### 4.3. Confidentiality

Data confidentiality is mostly achieved through encryption, using algorithms such as AES, DES, or RSA [60]. These algorithms are highly optimized and represent a mature technology, but often they require a conspicuous amount of processing power (it depends also on the parameters for encryption and the strength it is willing to achieve) [61]. While some years ago IoT devices were usually constrained in terms of processing power and memory [62], new devices take advantage of the technological advancements in the silicon industry that offer high processing power with little energy consumption. The new capabilities of embedded processors and microcontrollers allow advanced algorithms to be executed within the IoT device [63,64,65,66]. It is important to highlight that, in order to assure confidentiality, some encryption algorithms require the realization of a key exchange before opening a secure communication channel [67,68]. One of the most used silicon architecture in small devices is the ARM Cortex-M. Within these Integrated Circuits (ICs), it is common to find hardware accelerators for security applications, most notably the AES accelerator [69]. Running encryption algorithms in hardware allows very constrained devices, such as activity trackers, smart wearables, and RFID tags [70], to communicate confidentially, guaranteeing high levels of security.

### 4.4. Freshness

In some digital clinical trials that require delicate patient monitoring, the *delay* is a critical requirement. For example, for heart diseases such as arrythmia, identifying and generating early warnings require very short response times [65,66]. Existing IoT devices targeting healthcare suffer from a lack of computational power to locally process the ECG recordings and detect abnormal behaviour. Therefore, recorded signals need to be transferred to cloud services where advanced analysis algorithms are executed for processing and integration [71,72]. In other trials, the freshness requirement can instead be relaxed. For example, the glucose level in the blood can be delayed by minutes since it changes relatively slowly [73,74]. In typical IoT architectures, data from the IoT devices are transmitted to a nearby gateway device and then to cloud services for further processing, analysis, and integration [75]. Considering that the data flows follow different paths, it is natural to encounter delays, during disassembly and reassembly, as well as jitter in the communication [76,77].

### 4.5. Availability

Data storage solutions follow two main paradigms: *centralized* and *decentralized*. In *centralized* solutions, a single entity, such as a server or generally a device, stores all the data. While this can be convenient in terms of costs and the need for resources, it also makes the system less robust against hardware failures and power outages. In fact, a centralized system has a *single point of failure*. On the contrary, *decentralized* solutions allow for better scalability, do not suffer from a single point of failure, and are robust also against large-scale power outages. In IoT applications, it is common to find locally centralized systems [78,79,80,81,82] that send data to the cloud periodically, where the storage solutions are mostly decentralized [83,84,85,86,87,88]. This hybrid approach presents strengths such as ease of installation, low maintenance costs, and simple connection since a single device that acts as a gateway has to be configured and connected to the internet. However, this approach suffers a single point of failure and is prone to unavailability due to power outages. Nowadays, it is possible to realize solutions based on IoT devices without the need for a single and local gateway, exploiting communication infrastructures such as LoRaWAN [89,90,91,92] and SigFox [93,94]. The main limits of such architectures are the offered bandwidth and coverage [95] that are limited and possibly costly. Battery depletion causes unavailability. The research is trying to lower the power consumption with multiple approaches, from designing low power algorithms to changing in network topologies to implementing newer and more energy-efficient hardware. For example, in [96], the authors present a low-power system for acquiring and classifying biosignals coming from body sensors, while in [97] a mechanism is presented to adapt the radio power in order to decrease the overall energy consumption. In [98,99], the authors present a low power system capable of sampling, processing, and transmitting data for years. Despite the very fast improvement in silicon technologies, batteries do not follow the same trend and improve their capacity by about 5–8% every year [100].

### 4.6. Integrity

The concept of integrity is strongly connected to the *protection of information* from malicious third parties, cybercriminals, or any external interference from the initial transmission to the final reception of data. The systems must be aware of a threat whenever it tries to tamper with the data [101]. Malicious third parties could be interested in making revenue for their false outsourced data. A solution for such a problem is investigated in [102], where the authors provide an analysis of data integrity verification based on an authenticator suitable for both the cloud and the IoT. In [103], the authors present their solutions in order to achieve privacy preservation during the communications between all the components of an IoT system. In [104], the authors’ present public-key cryptosystems as desirable solutions whenever there is a need for data integrity and authenticity.

## 5. Characterization of Users and Recruiting of Participants in Trusted Space

In this section, we present the most common approach to address the requirements and technical challenges outlined in the previous sections, that is a centralized, possibly cloud-based *trusted authority* (the investigator in our use cases) that is responsible for the storage, control, and processing of the data of digital clinical trials. The data are collected by suitable IoHT devices both at home or elsewhere during different phases of a digital clinical trial. This data are then shared over a secure communication channel with the trusted authority that controls and stores it, possibly on a cloud-based infrastructure; this allows the investigator to exploit existing commercial platforms (e.g., such as AWS IoT (https://aws.amazon.com/iot/)) to accelerate the development process.

**Figure 2:**
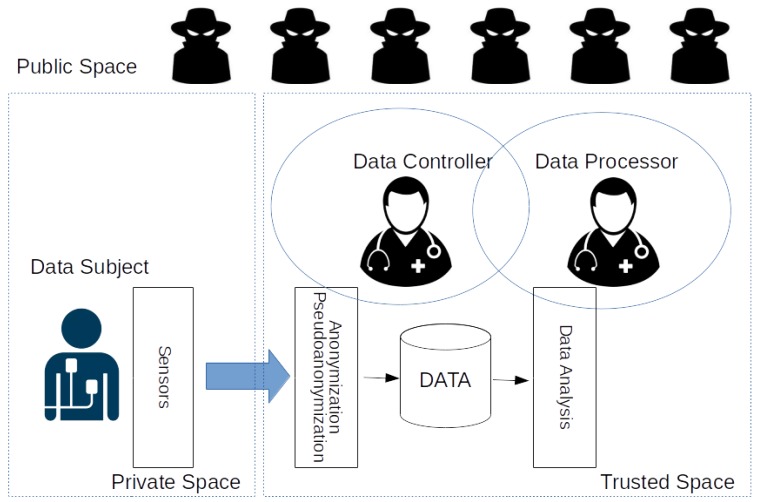
A simplified representation of the common approach in which according to Article 32 of GDPR anonymization and/or pseudoanonymization are in charge of the Data Processor and Data Controller.

A simplified representation of this process is shown in Figure 2, where three main domains are identified: the *private* space, the *trusted* space, and the *public* space. During all of the phases of the clinical trial, the data generated by the IoT devices in the *private space* is transmitted over a secure communication channel to the *trusted space*. Here, it can be enriched by other relevant data, possibly residing in the *public space*, such as gender, sex, and age. The *investigator* is operating within the trusted space and analyzes the data as an integral part of the research. It is evident that the user has severely limited control over the usage of its personal data as soon as it leaves its private space. It is therefore critical that the trusted space conforms to all regulations relevant to data protection. For this reason, the Data Controller and the Data Processor take care to anonymize or pseudonymize the data residing within the trusted space in order to be compliant with Article 32 of the GDPR (In this paper, for the sake of simplicity, the investigator assumes both the role of Data Controller and the Data Processor. In more complex scenarios, the Data Processor for clinical trials is the Sponsor).

We now introduce and analyze three techniques that can be employed to guarantee the anonymization or pseudonymization of personal data collected. We remark here there is an evident trade-off between the privacy that can be guaranteed to the participant and the usefulness of the data for the purposes of the clinical trial; the more the data is handled to preserve the privacy of the participant, the more difficult it becomes to extract information useful for the clinical trial. Note that the data in the public space are among the *“other information”* that can be used to tie pseudo-anonymized data to an individual patient.

### 5.1. k-Anonymity

*k-anonymity* is a practical approach for data anonymization. The starting point in k-anonimity is the idea that the features that would allow the identification of users are handled such that at least *k* records always exist in the dataset with the same set of features. Thus, it is difficult for an adversary to distinguish one specific record among those *k* records. In other words, for any given set of features, there exist at least *k* records in the dataset that contain them all. To clarify this concept, consider the example in Table 1. In this example, the adversary knows some information about the users, namely name, age, and ZIP. If the adversary can have access to the medical records, though they do not contain the name of the users, s/he can immediately correlate this information with the ones in her/his availability, to infer sensitive information about the disease of the users. Through *k*-anonimity (2-anonimity in this case) this risk can be reduced. Indeed, with this information, the adversary can claim that either Joe or Nic have Disease A (or B), but not exactly which one of them.

There is a clear trade-off between the re-identification probability that can be tolerated and the utility of data; while higher values of the *k* parameter imply a lower probability of re-identification, they also introduce more distortion to the data, in some cases reducing significantly the usefulness of the data. Reference [105] provides a survey on *k*-anonymity in data mining, while [106] explores the applicability of *k*-anonimity to health records. In the latter, the authors suggest that a hypothetical testing approach can be effectively used to control re-identification risk and to reduce the extent of information loss compared to baseline *k*-anonymity.

### 5.2. l-Diversity

In [107], the authors show two simple attacks to a *k*-anonymized dataset that can lead to severe privacy problems. The *homogeneity attack* exploits the limited diversity in some sensitive attributes. In particular, if the value for a sensitive attribute within a group of *k* records is the same, that value can be predicted exactly, even in a *k*-anonymized dataset. As an example of homogeneity attack, consider the case in Table 1, where the disease is C for both the records in the group with age [35,40] and ZIP [3,4]. The *background knowledge attack* relies on some background knowledge that might not be encoded in the dataset, but allows the attacker to infer the most likely values for some attributes. To overcome the homogeneity attack, the most simple definition of *l*-diversity [107] requires that the records in a group show at least *l* distinct values.

### 5.3. Differential Privacy

The main purpose of differential-privacy [108] is to make indistinguishable the output of an algorithm that analyzes a dataset and computes statistics, when a record in the dataset is either present or absent. In other words, looking at the output of the algorithm, one cannot tell whether any individual’s data were included in the original dataset or not. This implies that an adversary cannot learn anything (w.h.p.) about the presence or absence of that particular user, irrespective of the peculiar characteristics of that user. More formally, given a randomized algorithm *A* and two datasets D1 and D2 that differ in exactly one record (i.e., the data of one person), *A* is ϵ-differential private if for any S⊆Range(A)

$$Pr\left[A\left(D1\right)\in S\right]\leq e^{\epsilon }Pr\left[A\left(D2\right)\in S\right]$$

**Figure 3:**
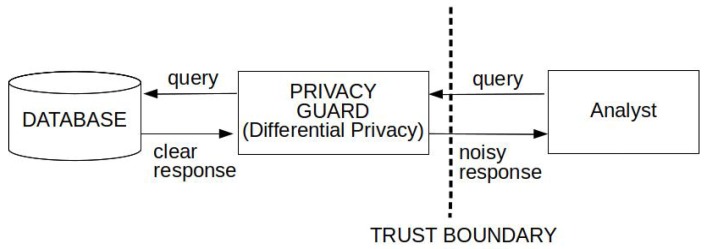
A simplified reference architecture.

The architecture of a differential privacy system [109] is represented in Figure 3. The analyst submits a query to the privacy guard, a software that assesses the privacy impact of the query “using a special algorithm”. The query is delivered to the database that responds. The guard adds some *“noise”* according to the evaluation of the privacy impact, and the noisy response is finally delivered to the analyst.

The correct evaluation of the privacy impact is crucial in this process and it is primarily related to the selection of the parameter ϵ, which is the parameter controlling the tradeoff between privacy and accuracy. While this is one among the most critical aspects for the applicability of differential privacy in practical cases, to the best of our knowledge, there is still no rigorous method to evaluate it in the literature. Dwork [110] indicates that the value of ϵ is a “social question”, leaving the problem de facto open, while in [111] the authors discuss the challenge of setting the proper value of ϵ given the goal of protecting individuals in the database with some fixed probability; they show that the clues about the fact that a specific individual is in the database or not can change depending on the query, on the values in the data, and even on values not in the data. More recently, the authors of [112] proposed a model that expresses the balance between privacy and accuracy, and they used such a model to choose ϵ on a series of simple statistical studies. Despite such efforts, still a satisfactory evaluation of ϵ is a challenge and it makes the applicability of differential privacy in practice difficult. Indeed, in the literature, the value of ϵ can range from 0.01 to more than 5. Finally, [113] analyses the main criticism of differential privacy. The paper [114] discusses the applicability of differential privacy to the healthcare domain. While the motivations supporting the use of differential privacy and the corresponding challenges have been very well explored, unfortunately the actual application of this technique in the real world healthcare is very limited. Very recently, the paper [115] has promised a step forward towards practical differential privacy for SQL queries. The authors implemented FLEX, a tool to enforce differential privacy for real-world SQL queries on any existing database with negligible performance overhead. Remarkably, the approach has been recently adopted by Uber to enforce differential privacy for their internal data analytics (https://medium.com/uber-security-privacy/differential-privacy-open-source-7892c82c42b6).

## 6. Characterization of Users and Recruiting of Participants in Private Space

When data management is left to a trusted authority (in our case the *investigator*), the user must fully trust it for the protection of her/his sensitive data. However, at least for the first phases of a digital clinical study (e.g., the characterization and recruitment of users, see Figure 1), it is possible to avoid the transfer of private data to the trusted space, nonetheless allowing the investigator to complete these phases without any access to confidential data [116,117]. The central idea is to take advantage of the increased computational capabilities of IoT devices and of the recent technological advancements in order to reinforce the privacy of confidential data in the *private space*, while still conforming to all requirements relevant to data protection, including the GDPR. Therefore, the user retains complete control over its private data and s/he is free to decide later if her/his data will be accessible by the investigator or not. At the same time, a private but useful sketch of the data is made available to the investigator that can still gain some knowledge over the population of users as a whole while ensuring the privacy of individuals. Obviously, in the subsequent phases, if the user will be actually enrolled in the trial, s/he will transfer the real data to the trusted space to allow the *investigator* to conduct the research. However, the investigator must apply all the necessary anonymization or pseudonymization steps required to comply with the regulations.

Such an alternative approach allows the users to retain the maximum control over their data until the *investigator* is certain that some value can be created from the use of their data, at which point the users will receive their fair part of the value created in exchange of their data. Therefore, the approach of moving data management from the trusted space to the private space follows the arguments of the *My Data is Mine* declaration (http://www.mydataismine.com/manifest).

The *investigator* has two main needs, as already discussed in Section 2, namely (a) characterize the community of potential users so that it can design an effective digital clinical trial and (b) recruit suitable users willing to participate. Both these tasks can be performed guaranteeing the privacy of the users.

**Figure 4:**
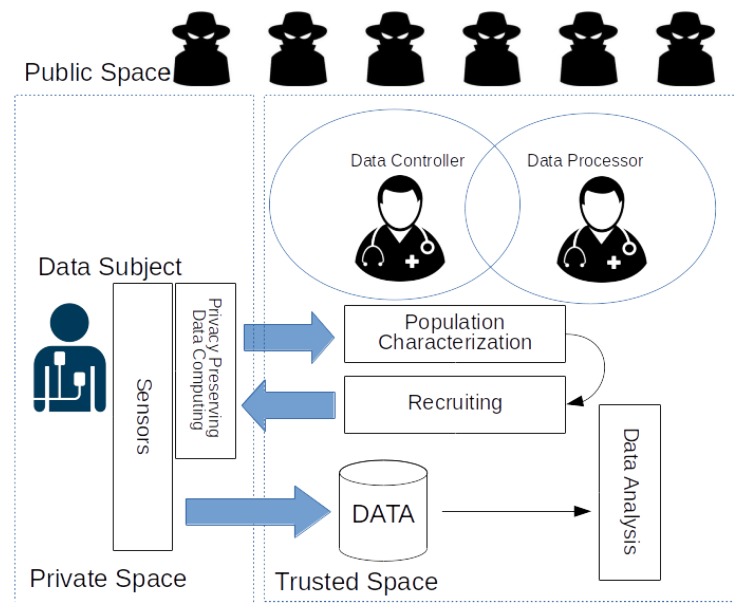
A simplified representation of the privacy-preserving characterization and recruiting phases of clinical trials. Once the user agrees to enroll, data are treated as in the common approach depicted in Figure 2.

A simplified representation of the proposed approach is shown in Figure 4. Initially, all of the interested users participate in the characterization process that allows the *investigator* to better design the digital clinical trial. After completing the design phase of the trial exploiting the insights distilled in the characterization phase, the *investigator* starts the recruiting process contacting all the users. Only those users that match the specific criteria and are willing to participate in the trial respond. In fact, the matching is computed in the private space of the users, so no personal information is disclosed before an agreement is reached. From the enrollment phase onwards, the trial follows the common approach discussed in Section 5. As a consequence, only users actually enrolled in the digital clinical trial will deliver their personal data to the trusted space, while all others will not reveal any relevant information except that necessary to characterize the population, a process that, however, has been designed to preserve privacy.

### 6.1. Proof of Concept

**Figure 5:**
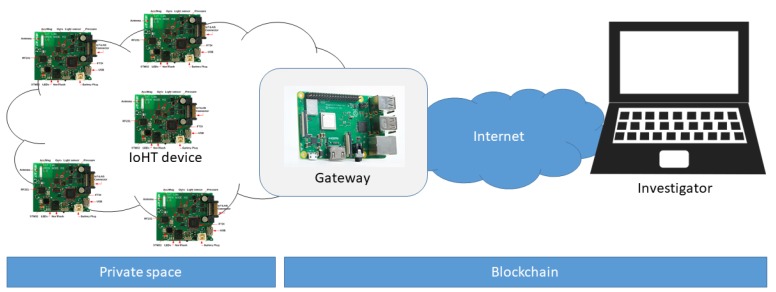
Experimental setup.

In the following sections, we present a proof-of-concept (PoC) of the proposed distributed data management approach depicted in Figure 4. The PoC implementation allows us to evaluate the proposed solutions on real-world hardware and obtain an initial feedback on the feasibility of this alternative approach. Our experimental setup is sketched in Figure 5.

In recent years, a number of new tools for managing large-scale IoT testbeds (e.g., see [118,119]) have been introduced. In order to implement a more realistic PoC, capable of demonstrating the interaction with a large number of devices in the private space, we interfaced our *gateway* with the IoT-Lab facility (IoT-LAB: a very large-scale open testbed, https://www.iot-lab.info/).

We employed different embedded devices available at the IoT-Lab to emulate different IoHT devices that generate biometric data. The testbed hosts devices with heterogeneous processors or microprocessors (such as Texas Instruments (Dallas, TX, USA) MSP430F1611 and SITARA AM3505 and ST Microelectronics STM32F103REY) that are equipped with wireless chips operating in the ISM (Industrial, Scientific, and Medical) radio spectrum. Most of them support the 802.15.4 protocol and operate at 868 MHz or 2.4 GHz. A Raspberry Pi3 (ARM Cortex-A53 64bits, RAM 1GB DDR2, Raspberry Pi Foundation, Cambridge, UK) is used as a generic gateway in the *private space* that collects all the data from the IoHT devices and communicates with the *trusted space*. The functionalities required by the *investigator* in the *trusted space* are implemented on a standard PC (Intel i7-6500U, RAM 8GB DDR3, Intel, Santa Clara, CA, USA). The gateway and the PC interact with the Ethereum blockchain to guarantee to the *investigators* the quality of the data provided by the users (see Section 2).

In the following sections, we run our experiments to evaluate the performance of the PoC to (a) guarantee the originality and authenticity of the data collected in the private space (see Section 6.2), (b) characterize the community of potential participants (see Section 6.3), and (c) recruit suitable patients for the trial (see Section 6.4).

In order to asses the feasibility of our solution on the proposed PoC, we compared the performances of the considered algorithms running on the gateway (i.e., a constrained device typical of IoHT deployments) and on a standard PC.

### 6.2. Guaranteeing Originality and Authenticity of Collected Data

The requirements for the data collection phase were analyzed in Section 2 and can be summarized in the following points.

- The quality of data provided by devices must be verifiable.
- Data are collected by certified and trusted devices.
- Fake data cannot be introduced into the private space.

We suppose that an external entity conducted accuracy evaluation tests over various off-the-shelf IoT devices (wearables, smart devices, etc.) and compiled a list of “trusted” devices that are accurate enough for the use in healthcare applications. Only the data collected from these devices can be used in digital clinical trials.

One way to guarantee the integrity, authenticity, and accuracy of the data gathered from IoHT devices is through the use of an asymmetric encryption scheme, signing every chunk of data, before sending it to the *gateway*. Thus, all packets sent from IoHT devices to the gateway include measurements that are signed. The *gateway* can check their integrity and authenticity, and discard those that are arriving from untrusted devices or with a bad signature. Moreover, the digital signing is done over measurements that include an identifier that encodes the manufacturer, model number, and production series along with a private key that is installed in a tamper-proof memory space, while the corresponding public key is stored in the blockchain residing in the *public space* to make it publicly accessible by both the *gateways* and the *investigators*. Therefore, the *investigator* can identify the devices that generated data for each individual user, and it can decide if the data received after the enrolling are within the accuracy requirements.

In our PoC implementation, we used the open-source uECC library (https://github.com/kmackay/micro-ecc) available on RiotOS (https://riot-os.org/) to sign the data. The software ran in the *M3 open nodes* at the IoT-Lab premises. This implementation supports the recommended elliptic curve [120] over binary fields with equation $y^{2}+xy=x^{3}+x^{2}+1$ along with the irreducible polynomial $f\left(x\right)=x^{163}+x^{7}+x^{6}+x^{3}+1$. The order of the curve (the number of points on it, *r*) and the base point G(x,y) are listed in Table 2.

The blockchain technology provides a *distributed ledger* that is highly tamper-resistant and immutably records every action executed on the chain. This technology allows us to guarantee the integrity of the data using hash functions. In our PoC, we used the *Ethereum* Blockchain App Platform (https://www.ethereum.org/). The gateway stores periodically the hash of data received from the IoHT devices in the blockchain. Note that no private data are stored in the chain—only their hashes that allow the investigator to check the integrity and authenticity of the data.

**Figure 6:**
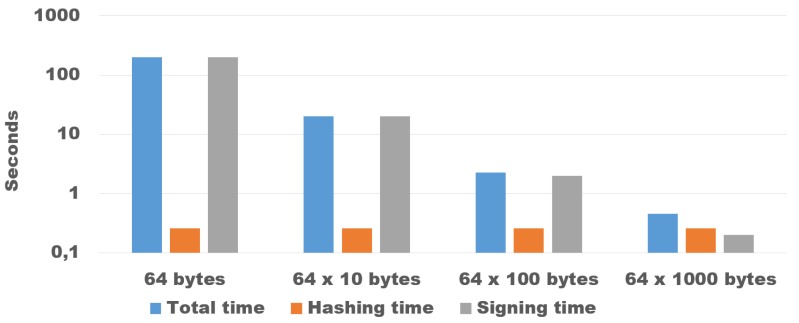
Time necessary to sign chunks of data of different sizes for an overall of 64,000 bytes. Please note the logarithmic scale.

Our evaluation shows that the signature function is by far the most time-consuming, while the hashing is always relatively fast. Figure 6 shows the time necessary to sign 64,000 bytes of data on the resource constraint *M3 open nodes*, dividing them in chunks of different size (1000×64 bytes, 100 × 640 bytes, 10 × 6400 bytes, and 1 × 64,000 bytes). The total time necessary to sign 1000 chunks of 64 bytes were more than 200 s, namely more than 400 times bigger than the time necessary to sign the chunk of 64 Kbytes (about half a second). Furthermore, when we consider chunks of 64 bytes, the signature occupies about one-third of the payload, while in the 64,000 bytes case it is less than 0.1%. This result suggests that some form of aggregation is always necessary to implement a practical solution.

### 6.3. Characterization of Potential Participants

The *investigator* is interested in grouping candidates according to their characteristics in order to understand the community of potential patients, and consequently better design the clinical trial. For this purpose, a *privacy-preserving data clustering* is conducted by carefully specifying the desired features relevant for the purpose of the trial. These features include the evaluation of specific biometric attributes (body composition, heart operation, daily activity, etc.) collected from the patient using IoHT devices over a period of time (blood pressure for the past week, etc.). During this process, the privacy of the users is preserved since none of their private data exit from the private space. The only information sent back from the institute are the ones necessary to identify the clusters without disclosing data of a single user or its cluster membership.

**Figure 7:**
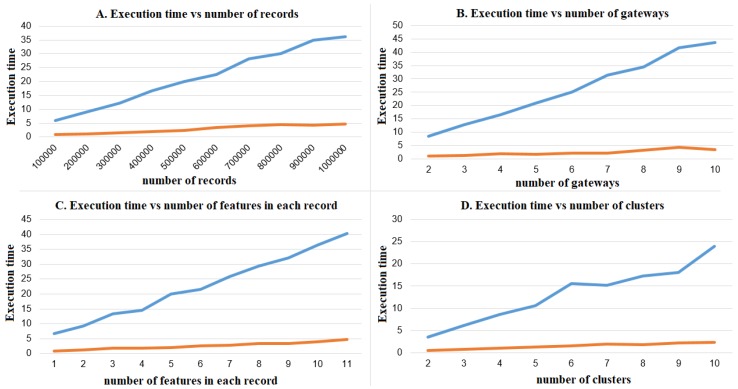
Execution time (*x* axis of all four plots, in seconds) of the privacy-preserving clustering algorithm on PC (orange) and *gateway* (blue), in (**A**) for a different number of records (*y* axis, the number of records), in (**B**) for a different number of gateways (*y* axis, the number of gateways), in (**C**) for a different number of features (*y* axis, the number of features in each record processed by the clustering algorithm), and in (**D**) for a different number of clusters (*y* axis, the number of clusters).

For the sake of simplicity, horizontally distributed data are considered in which the personal data of each party are disjoint and the parties want to jointly cluster their records without revealing their personal data. In the experiments reported here, the library presented in [121] is used to implement the privacy-preserving *k*-means algorithm proposed by Samet and Miri [122]. We want to evaluate to what extent the proposed algorithm can run on the resource constrained gateway and to what extent the performance degrades with respect to a more powerful standard PC. The results are shown in Figure 7.

Four parameters are examined independently: the number of records collected by the IoHT devices (Figure 7A), the number of gateways participating in the privacy-preserving computation (Figure 7B), the number of features composing each record (Figure 7C), and the number of clusters (clusteroids) used as input (*k*) to the clustering algorithm (Figure 7D). The effect of each of these parameters is evaluated independently by keeping the others fixed. However, it is clear that these parameters affect the overall performance of the algorithm. We used a synthetic dataset containing feature values that are obtained from realistic distributions of biometric data, such as heart rate, body mass, and steps. Results are obtained by averaging 10 iterations of the algorithm.

The experimental results show that the the gateway and the PC execution time are both linear for all the considered parameters, but the slope of the gateway performance is significantly steeper even if there is no evidence of saturation in the considered scenarios. The observed computation times are acceptable, in the order of tens of seconds, even if in this experiments the latency due to the network communications has not been considered.

### 6.4. Recruiting Patients

The *characterization phase* allows the *investigator* to better understand the population of potential participants. On the basis of this knowledge, it can design a *recruiting test*, namely a piece of software that is executed on the gateway in the private space of the user, taking as input an adequate set of genuine historic values collected from one or more IoHT devices and providing as output a boolean value indicating whether or not the user is suitable for the trial. Since the recruiting test is executed in the private space, no private information of the users are disclosed to the investigator or elsewhere outside the private space (This idea of algorithm to the data (not vice versa) has been indicated by MIT media lab as one of the OPEN TrialChain key principles).

The result of the recruiting function is then presented to the user who can agree to participate in the trial (proceeding to the enrollment phase and thus delivering her/his data to the trusted space) or can reject participation (where no personal data leave the private space).

The requirements for the recruiting phase have been analyzed in Section 2 and can be summarized in the following points.

- The inclusion/exclusion of a candidate is based on the result of a well-defined *recruiting test* automatically executed in the private space over a data set of genuine historic values.
- An individual that wishes to be considered for a specific clinical trial expects that the privacy of her/his personal data will be respected and the confidentiality of the private data will be guaranteed. If during the recruiting phase the individual is excluded, then the digital health system should guarantee that no personal data are retained by the *investigator*.

We consider two distinct classes of *recruiting tests* used for inclusion/exclusion. A “basic” one that covers nowadays common selection criteria and an “advanced” one that could cover future criteria.

- The class of *basic recruiting tests*, where the *recruiting test* receives as input the raw data in the private space, possibly pre-processing it to extract relevant features. As an example, the recruiting test can be a threshold on the average heart-rate.
- The class of *advanced recruiting tests*, where a machine learning algorithm on the gateway is trained with the raw data in the private space as the *training set*. The *recruiting test* is computed over the output of the trained machine learning algorithm receiving as input a *test set* provided by the investigator.

In order to evaluate the performance of these two classes of recruting tests, four real-world datasets available in the UCI Machine Learning Repository [123] were used. They vary both in the number of samples and in the number of features, accordingly to Table 3. Two of them target health issues as cancer and heart diseases (*Arcene* and *Heart Diseases—Cleveland*). *EEG Eye State* correlates the EEG with open or closed eyes, while *Gisette*, due to its size, has been selected to stress the performance analysis.

**Basic recruiting tests.** Raw data generated by the IoT devices in the private space are elaborated in order to extract relevant features provided as input to the *recruiting test*. In this experiment, the ECG data are analyzed following the methodology of [124]. First, a *Butterworth Band Pass Filter* of the fifth order is applied. Then the *fast Fourier transform (FFT)* and the associated *power spectral density (PSD)* are calculated. Finally, the mean, the standard deviation, the variance, and the maximum peak of the signal are computed for statistical purposes.

Table 4 reports the execution times required to analyze one hour of real ECG data sampled at 360 Hz [125]. The findings indicate that the *gateway* is roughly an order of magnitude slower than the *PC*, but it can extract complex features in absolutely reasonable times.

**Advanced recruiting tests.** The main goal of this experiment is to evaluate the ability to support the computation of complex *advanced recruiting tests*. Six well-known machine-learning algorithms available in the *Python* package *scikit-learn* [126] have been considered. Their execution times are shown in Table 5. We remark here that the objective of such an evaluation is to measure the performance in terms of execution time. Since both processing units are executing the same algorithms with exactly the same implementation, the accuracy is the same. The fine-tuning of such algorithms, in order to improve the effectiveness of the results, is not within the scope of this work.

In all cases, the *gateway* is an order of magnitude slower than the PC, but it can execute the algorithms within a reasonable time. The algorithms that are more demanding in terms of memory requirements can easily exhaust the available RAM memory, generating significant delays (e.g., due to memory swaps) and in some cases they create *Memory Errors* on the resource-constrained *gateway*.

## 7. State of the Art

As stated in Section 1, the healthcare system is transforming itself to exploit the potentialities of IoT. While the IoHT technologies offer some benefits, they also pose new challenges. For example, the work in [127] validates the effectiveness of using IoT devices in the follow-up of diabetes outpatients, while in [128] it is recognized that current personal health data are prone to hacking because of security vulnerabilities. From the studies in [129], it is clear that IoT will soon revolutionize the healthcare system. Several studies have introduced IoT in the healthcare domain [130]. However, security threats threaten to refrain the development of smart health applications in large-scale heterogeneous scenarios. One interesting approach to solve this problem is presented in [131], where a flexible security enforcement framework is proposed along with a policy definition language that enables the definition of cross-domain policies in order to face security and quality threats in dynamic large-scale and heterogeneous smart health environments. Another approach is through the use of blockchain technologies that open new possibilities in the field of healthcare. For example, in [132], the authors propose a platform to conduct trials and better support precision medicine. In [133,134], the authors propose social-media-based approaches to raise awareness of clinical trials. These solutions greatly reduce the cost of advertising (often done through other media, such as newspapers, television, and radio) but do not help in characterizing the population before the recruitment phase. Exploiting social networks can allow researchers to easily reach a wide audience. To overcome the difficulties in recruiting adolescents, the authors in [135] propose a mixed approach of social media use (Facebook) with traditional paper mailing. On the other side, the inclusion of people over 90 years old is of particular interest in clinical research; in fact, they compose the fastest growing segment of the population [136]. In the *90+ study*, one of the inclusion criteria was that the individuals must be within a one-hour drive from the study location. This limit can be exceeded using remote monitoring system or similar solutions offered by eHealth. The authors in [137] applied the MARKIT (Marketing and Information Technology) model to the SMART study (a clinical trial of weight loss for college students). Some of the subjects in the SMART study were monitored using IoHT devices. In the work of [137], all data were collected within a single system, where study staff could monitor, for example, the completion of questionnaires and more, with a clear implication of privacy. The authors in [138] proposed a high-level view of their architecture to efficiently use wearable IoT in healthcare. From their study, the need for standards and regulations emerged. The work [139] proposes the submission of online personality questionnaires in order to increase the efficiency of recruitment. This additional step helps to identify the potential participants who will meet key criteria. The authors in [140] propose an architecture to collect and process health data produced by specialized IoT devices. Their approach uses a centralized structure with an added privacy-preserving and security enforcement module at the edges. They do not dive, however, into the recruiting process of digital clinical trials. The article [141] highlights the importance of wearable devices in both recruiting participants for digital clinical trial and the successive follow-up (in following strict treatments but also in the long run).

It is crucial to keep in mind that smart wearables integrate sensing, computation, and wireless communication in small, low-power devices that in many cases may operate in uncontrolled environments. Such low-sized embedded devices have limited sensing, signal processing, and communication capabilities and are usually battery-operated. Due to this resource-constrained environment of operation, applying standard security and privacy requirements is extremely challenging [142]. As an example, consider that some smart devices have limited computing and storage capabilities, thus cryptographic algorithms and protocols that require intensive computation, communication, or storage are simply not applicable. It is too costly (in terms of computation) to authenticate using a public key and too costly (in terms of memory and computation) to store one-way chains of keys. Consider also that some smart devices may be battery-operated, forcing security mechanisms to reduce their energy consumption. These constraints greatly increase the difficulty of securing IoT-enabled systems and make them more vulnerable to security threats [143,144,145].

A key technical component of our solution in the *characterization of the population* phase is privacy-preserving computation [146], namely a set of elaborate techniques that transform users’ private data to protect users’ privacy and still maintain a good level of accuracy when exploring and analyzing the data. In our PoC, we exploited the work of [122], but a number of other solutions can be applied. One of these is differential privacy [108,110], which was introduced in Section 5, and the applicability of which has been already investigated in the context of clinical trials in [147]. Other interesting techniques project the original data in a reduced space, trying to reducing the complexity of problems, while maintaining the usefulness of the projected data for algorithms and guaranteeing some level of privacy for the users. In [148], the authors proved that the Johnson–Lindenstrauss transform can be used as an alternative approach to achieve differential privacy. Random projections (RPs) can provide useful data for machine learning algorithms on a group of potential patients, while preserving at the same time the privacy of individuals. Furthermore, they have been already employed in a number of healthcare applications, such as to classify cancer [149]. In [150], RPs are used to mask clear data, projecting them in smaller spaces, whereas, in [148,151], the authors discuss how RPs can be exploited to enhance data privacy.

## 8. Conclusions

In an increasingly data-driven world, where information sharing, machine learning, and social networking leads the way, the IoT will be a key technology for person-centric mobile e-health. This work looks into the usage of IoT technologies as an integral part of clinical trials so that data residing in an IoT world can enable drug developers to obtain better insights and streamline the overall clinical trial processes.

The current dominant approach for the management of health data in clinical trials requires that users trust a third-party, the investigator, that handles their data for the purposes of the trial. In the first phases of a digital trial, the main purposes of the investigator are (a) the characterization of the population of potential participants in the trial, as this investigator needs to know the amount of users potentially interested in participating (and fit to participate) in the trial, and (b) the effective recruitment of patients.

In this paper, we investigate a solution that, at least for these first phases of a clinical trial, can protect the interests of both the investigator (i.e., the utility of the data) and the participants (i.e., the privacy of the data). Indeed, the original data never leave the private space of the patient during these phases. Only suitable sketches of the data, able to support the purposes of the investigator, are delivered, while the proofs on the quality and authenticity of the collected data are stored in the blockchain in the form of hashes. Our proof-of-concept of the proposed solution shows that it can be effectively implemented with a fairly reasonable performance on nowadays resource-constrained devices typical in IoT deployments.

As a future work, we plan to extend the characterization of the population of users employing other private computation techniques such as differential-privacy and random projections. 

## Figures and Tables

**Table 1:** On the right, the adversary’s knowledge; in the center, the original medical records; on the left, a 2-anonymous table.

Name	Age	ZIP	Age	ZIP	Disease	Age	ZIP	Disease
Joe	15	1	15	1	A	[15,18]	[1,2]	A
Nic	18	2	18	2	B	[15,18]	[1,2]	B
Lou	35	3	35	3	C	[35,40]	[3,4]	C
Mary	40	4	40	4	D	[35,40]	[3,4]	D

**Table 2:** Parameters for the Basic Elliptic Curve Operations.

Parameter	Value
*r*	0x4000000000000000000020108a2e0cc0d99f8a5ef
*x*	0x2fe13c0537bbc11acaa07d793de4e6d5e5c94eee8
*y*	0x289070fb05d38ff58321f2e800536d538ccdaa3d9

**Table 3:** Datasets used to evaluate performances.

#	Dataset Name	Number of Samples	Number of Features
1	Arcene	100	10,000
2	EEG Eye State	13,444	14
3	Heart Disease	270	13
4	Gisette	6000	5000

**Table 4:** Execution times for ECG analysis.

	PC	Gateway
**Filtering**	0.114648 s	1.483026 s
**FFT and PSD**	0.621372 s	4.410873 s
**Statistics**	0.016048 s	0.115040 s
**Total**	0.752068 s	6.008939 s

**Table 5:** Execution times of common machine learning algorithms. Each row corresponds to the equivalent dataset in Table 3.

	#	PC	Gateway
Support Vector Machines	1	0.147322 s	0.695988 s
	2	45.561 s	565.288 s
	3	0.005111 s	0.061623 s
	4	250.205 s	1180.824 s
Logistic Regression	1	0.133114 s	1.639809 s
	2	0.177369 s	2.124157 s
	3	0.001498 s	0.020984 s
	4	1.707945 s	22.940 s
k Nearest Neighbors	1	0.010120 s	0.086751 s
	2	0.009555 s	0.125092 s
	3	0.000355 s	0.002438 s
	4	1.315992 s	18.851 s
Gaussian Mixture Models	1	0.245765 s	2.103226 s
	2	0.667314 s	7.934804 s
	3	0.011209 s	0.087351 s
	4	30.658 s	Memory Error
k-Means	1	0.116964 s	0.859489 s
	2	0.029887 s	0.293893 s
	3	0.003146 s	0.035679 s
	4	8.616173 s	Memory Error
PCA	1	0.463008 s	3.250940 s
	2	0.055881 s	0.662027 s
	3	0.000539 s	0.003660 s
	4	40.488 s	Memory Error
